# Psychometric Properties of the Autoquestionnaire Qualité De Vie Enfant Imagé (AUQEI) Applied to Children with Cerebral Palsy

**DOI:** 10.1371/journal.pone.0115643

**Published:** 2015-02-11

**Authors:** Wener Barbosa-Resende, Viviane de Oliveira Rangel, Ana Claudia Frontarolli, Renata R. Hoffman Araújo, Carlos Henrique Martins da Silva, Rogério de Melo Costa Pinto, Nívea de Macedo Oliveira Morales

**Affiliations:** 1 Graduate Program in Health Sciences, Faculty of Medicine, Federal University of Uberlândia, Uberlândia, Minas Gerais, Brazil; 2 Faculty of Medicine, Federal University of Uberlândia, Uberlândia, Minas Gerais, Brazil; 3 Physical Therapy and Specialty Center Unimed, Uberlândia, Minas Gerais, Brazil; 4 Association for Assistance to Disabled Children,Uberlândia, Minas Gerais, Brazil; 5 Department of Pediatrics, Graduate Program in Health Sciences, Faculty of Medicine, Federal University of Uberlândia, Uberlândia, Minas Gerais, Brazil; 6 Center of Exact Sciences and Technology, Faculty of Mathematics, Uberlândia, Minas Gerais, Brazil; University of Toronto, CANADA

## Abstract

**Background:**

Quality of life (QL) assessments of children with incapacitating diseases, such as cerebral palsy (CP), have often been conducted with the help of the representatives of a child, making QL assessment more subjective. TheAutoquestionnaireQualité de Vie Enfant Imagé (AUQEI) is a QL assessment designed for children to self-report—it uses images to facilitate the reporting process.

**Objective:**

evaluate the psychometric properties of AUQEI when responses are given by children with CP.

**Findings:**

Children aged 4 to 12 years (45 with CP and 45 healthy children) gave responses to the questionnaire. The data quality, reliability and validity were assessed. The data loss rate ranged from 8.8% to 46.7%, and was highest for the “autonomy” factor. No floor or ceiling effect was detected. The success rate for reliability of the internal consistency of the items was less than 80% for the “autonomy” factor. Cronbach’s alpha coefficient was 0.71 for the instrument and less than 0.5 for the factors. All the factors had a success rate of greater than 80% for the discriminating validity of the items. The factors did not have correlations between each other, thus indicating adequate discriminating validity. Convergent validity was tested and a significant correlation was demonstrated only between the AUQEI “functioning” factor and the Child Health Questionnaire—50-Item (CHQ-PF50) physical summary score (r = 0.31, p = 0.042). The AUQEI scores did not have correlations with the gross motor function scores (p>0.05) as expected for divergent validity. Regarding construct validity, the total AUQEI score obtained by the CP group was lower (median: 47.3) than that of the healthy group (median: 51.0) (p<0.01).

**Conclusion:**

The AUQEI was shown to be a reliable and valid instrument for assessing children with CP when the total score was used. Convergent validity should continue to be tested in future studies.

## Background

The assessment of quality of life (QL) in children with cerebral palsy (CP) is important because this population has reduced activity levels and limited participation due to disorders of movement, posture, sensation, perception, cognition and communication imposed by the disease, which can lead to negative repercussions in QL [1], [2]. According to the World Health Organization Quality of Life (WHOQOL), QL is defined as “the individual’s perception of their position in life, in the cultural context and value systems in which they live and in relation to their goals, expectations, standards and concerns” [3].

The QL studies among children with incapacitating diseases such as CP [4] present limitations with regard to responses obtained directly from the patients and there is often a need to rely on a representative [5]-[7]. Although this means of obtaining data is indispensable in situations of greater cognitive impairment, it makes QL assessments more subjective [8]. Because of differences of perspective between children and their parents [9], efforts towards refining and placing value on measurements that evaluate children’s own perceptions have increasingly been made. Some generic instruments such as the Pediatric Quality of Life Inventory (PedsQL) and the European generic Health-related Quality of Life questionnaire (KIDSCREEN) [10], [11] and specific instruments such as the PedsQLcerebral palsy module and the Cerebral Palsy Quality of Life Questionnaire for Children (CPQOL-Child)[10], [12], have been validated to be answered by children with CP themselves.

A systematic review evaluated the psychometric properties and clinical utility of all condition-specific outcome measures used to assess QL in school-aged children with CP, and the Caregiver Priorities and Child Health Index of Life with Disabilities (CPCHILD) and the CPQOL-Child demonstrated the strongest psychometric properties and the greatest clinical utility [2]. Another study compared the conceptual differences, internal consistency, and validity of three QL questionnaires. Conceptually and psychometrically, KIDSCREEN-10 and CPQOL-Child performed more strongly than the Child Health Questionnaire (CHQ) for children with CP. The choice between these instruments will depend on the questions posed and outcomes sought by the researcher or clinician [1]. But it is important to consider the psychometric properties of the instruments and if it is applied directly to the child or to a proxy.

The AutoquestionnaireQualité de Vie Enfant Imagé(AUQEI) is a generic QL instrument that has the advantage of facility of use—it utilizes the support of images so that children can better express their comprehension of the questions and their satisfaction regarding family factors, social factors, activities, health, body functions and separation. This instrument is easy to apply and can be answered from the age of four years onwards [13], [14]. It has also been applied to children with CP, although its psychometric properties have not been tested for this population[15]-[17]. Hence, the objective of the present study was to evaluate the psychometric properties of AUQEI when responses are given by children with CP.

## Methods

### Participants and procedure

Over a six-month period, children with CP aged 4 to 12 years who frequently visited a pediatric rehabilitation unit (Association for Assistance to Disabled Children-Minas Gerais/AADC-MG) were invited to participate in a cross-sectional study. Informed consent was obtained in writing from parents, caregivers or guardians of the children who participated in the survey. Children with cognitive difficulty as measured using the Wechsler Intelligence Scale for Children, Third Edition(WISC-III), were excluded[18]. The healthy group was formed by children of the same age group without chronic diseases.

The patients were evaluated by a neuropediatrician to confirm the diagnosis of CP and the clinical data; by a physiotherapist to evaluate physical functioning (Gross Motor Function Measure—GMFM, and Gross Motor Function Classification System—GMFCS)[19], [20]; and by a psychologist to make a cognitive assessment (WISC-III)[18].

The children with CP were classified according to the most dominant clinical characteristics and by anatomic distribution of the lesion. In accordance with changes of muscle tone and movement, the children were classified into the following types: spastic (presence of weakness, hypertonia, hyperreflexia, clonus and plantar cutaneous reflex in extension—positive Babinski, caused by injury to the pyramidal system)[21], [22]; dyskinetic (evident abnormal movements, caused by inadequate regulation of coordination and muscle tone, includes rigidity, chorea, choreoathetosis, athetosis and dystonia, occasioned by a lesion of the extrapyramidal system)[23], [21]; and ataxic (disturbance of coordination of voluntary movements because of dyssynergia, caused by a dysfunction in the cerebellar)[23], [24]. And according to the anatomical distribution, the patients were divided into the following categories: quadriplegia (involves all four limbs and trunk)[21]; diplegia (lower limbs are affected more severely than the upper limbs)[21]; and hemiplegia (impairment of a hemibody with more severe involvement of the upper limbs than the lower limbs, in general)[21]. Intellectual disabilities are present in almost all cases of quadriplegia in the literature[21].

Social, demographic and educational level data were obtained from caregivers. The children were invited to answer the AUQEI (on interview) during an interview with a psychologist. Parents answered two health-related quality of life instruments (HRQL): the initial Brazilian version of the Child Health Questionnaire—50-Item, Parent Complete Short Form (CHQ-PF50)[25], [26] and the Childhood Health Assessment Questionnaire (CHAQ) Brazilian-version[27], [28].

The sample size was estimated from the prevalence data of the CP [29] and from the data published in 2003 by the Center of Studies, Research and Economic-Social Projects (CSRESP) of the Institute of Economics of the Federal University of Uberlândia (UFU) [30].

The population of the study (N) was calculated from the average number of live births in the city of Uberlândia—Minas Gerais—Brazil, in the years of 1998 (n = 7885) and 1999 (n = 8875) [30], (last two years of birth of inclusion criteria—age), resulting an average of 8380 individuals. The average of live births, between the years 1998–1999, was multiplied by 8, finding a population of 67040 children births between 1991–1999, aged 4–12 years in 2003, (age at which children answered the questionnaire AUQEI). Thus, according to the prevalence of CP (2.18/1000 live births) [29], the size estimated of the population of children with CP live births in Uberlândia—Minas Gerais—Brazil, between the years 1991–1999, was of 146.1472 children.

The sample of the study (n) was calculated from the estimated number of the population of children with CP between 4–12 years old, in Uberlândia—Minas Gerais—Brazil—2003, considering a prevalence of 0.218% [29], with the confidence of 99% and error of 1.5% [31].

This study was approved by the institutional review board (AACD-MG 66/2003 and the Federal University Uberlândia-UFU182/03).

### Instrument

**Autoquestionnaire Qualité de Vie Enfant Imagé (AUQEI)**

AUQEI is a generic questionnaire for self-assessment of QL[13] that has been translated into Portuguese, culturally adapted and validated for healthy children[14]. It provides a perception of the well-being of children aged 4 to 12 years through using 26 questions with four alternative responses. These responses can be visualized as four images of faces that express different emotional states relating to different domains of the child’s life.

The scale is composed of four factors (autonomy, leisure, functioning and family). Questions 6, 7, 9, 12, 14, 20, 22 and 26 are not included in these factors because they represent domains that are separate from the others.

The score for each item ranges from 0 to 3, and higher scores signify better QL. The scale makes it possible to obtain a single score resulting from summing the scores attributed to each item.

### Statistical analysis

Descriptive statistical analysis was used for the demographic and clinical data. The proportion of questionnaires with missing data and floor and ceiling effect were calculated for each factor.

The missing data refer to the proportion of participants who did not complete at least one item of the factor and were considered present when they exceed 20% [32]. The floor and ceiling effect are defined, respectively, as the proportion of patients who had the lowest and highest possible scores for each factor evaluated, and were considered present when they exceed 10% [32].

Internal consistency reliability was verified using Cronbach’s alpha coefficient [32].

Item internal consistency and item-discriminant validity were assessed and considered satisfactory if the success rate of the factors was higher than 80% [32].

Discriminant validity was tested by the correlation between all factors. A weak correlation was expected [33].

Divergent validity was determined through the correlation between the AUQEI with the GMFM. A poor correlation was expected[33].Convergent validity was determined by the correlation of the AUQEI with the CHQ-PF50 physical and psychological summaries and the CHAQ disability index. A significant correlation was expected (p< 0,05).

The Spearman correlation coefficient was used for all correlation tests.

To assess construct validity, the Mann-Whitney t-test was used to compare the scores of the CP group with the group of healthy children.

## Results

Out of the 73 patients who were eligible for the study, 28 (38.9%) were excluded because they presented cognitive limitations that made it impossible to answer the questionnaire. Thus, 45 children with CP participated in the study. Their mean age was 8.1 years (SD: 2.2) (Table 1). The healthy group was composed of 45 children with a mean age of 7.5 years (SD: 1.8), of whom 55.6% were female, and 100% of them were in regular school.

**Table 1:** Demographic and clinical characteristics of children with cerebral palsy.

Characteristics	Value (n = 45)
Mean age (years) (SD)	8,1 (2.2)
Male (n) (%)	25 (55.6)
Ethnicity (n) (%)	-
- Caucasian	45 (100)
Classificationof CP (n) (%)	-
- spastic	41 (91)
. quadriplegia	2 (4.4)
. diplegia	19 (42.2)
. hemiplegia	20 (44.4)
- extrapyramidal/dyskinetic	1 (2.2)
- ataxic	3 (6.7)
GMFCS (n) (%)	-
- level 1 and 2	31 (68.9)
- level 3	11 (24.4)
- level 4 and 5	3 (6.7)
GMFM—mean of end score (SD)	78,15 (24.58)
Epilepsy (n) (%)	13 (29.5)
Education (n) (%)	-
- not receiving education	0
- receiving special education	5 (11.1)
- receiving regular education	40 (88.9)

SD = standard deviation

CP = Cerebral Palsy

GMFCS = Gross Motor Function Classification System

GMFM = Gross Motor Function Measure

### Psychometric properties of AUQEI

**Data quality**

The data loss rate ranged from 8.8% to 46.7% for the factors (Table 2). Out of the 1170 questions, 94 were not answered. The items with the fewest responses were the following question numbers: 3 (10 patients responded), 14 (20 patients), 17 (16 patients), and 24 (20 patients) (Table 2).

**Table 2:** Quality data, reliability and item internal consistency.

AUQEI-Factors	Data Quality			Reliability		Validity
	Missing data(%)	Floor effects (%)	Ceiling effects (%)	Internal consistency reliability^a^	Item internal consistency^b^ (% success)	Item discriminant validity^b^ (%success)
Function	8.8	0	0	0.0529	80	95
Family	26.7	0	2.2	0.4872	100	100
Leisure	8.88	0	4.4	0.1416	100	100
Autonomy	46.7	0	0	0.2454	40	90

^a^Cronbach’s alpha coefficient

^b^Spearman’s correlation coefficient

The floor and ceiling effect rates are presented in Table 2.

**Reliability**

Regarding the internal consistency of the items, the success rate was less than 80% for the factor “autonomy” (Table 2). Cronbach’s alpha coefficient was 0.71 for the instrument, and it ranged from 0.05 to 0.49 for the factors (Table 2).

**Validity**

The success rate for the discriminating validity of the items was greater than 80% for all the factors (Table 2).

In determining correlation between the factors, only the “functioning” factor presented correlations: with family (r = 0.47) and with autonomy (r = 0.41) (p < 0.01).

There was no correlation between the AUQEI scores and the GMFM (p>0.05). The CHQ-PF 50 physical summary score correlated with the AUQEI “functioning” factor (r = 0.31; p = 0.042) but it did not correlate with the others factors or the total score of the AUQEI (p > 0.05). There was no correlation between the AUQEI scores and the CHQ-PF 50 psychosocial summary or the CHAQ disability index (p>0.05).

The patients presented lower total AUQEI scores than shown by the healthy group (p = 0.001)(Table 3).

**Table 3:** AUQEI scores obtained by patients and healthy group, in assessing construct validity.

Factors	Patients(percentiles 25–75) (n = 45)	Healthy group(percentiles 25–75) (n = 45)	p value*	Effect Size
Function	10.0 (8.5–11.0)	10.0 (8.5–11.0)	0.803	-
Family	10.0 (8.0–10.0)	12.0 (10.0–13.0)	0.000*	0.67
Leisure	6.0 (6.0–7.0)	8.0 (7.0–9.0)	0.000*	1.00
Autonomy	6.0 (5.0–7.5)	8.0 (5.0–9.0)	0.036*	0.50
Total	47.0 (42.0–50.5)	51.0 (48.5–53.0)	0.001*	0.89

*Mann-Whitney’s test

## Discussion

The present study indicated that the psychometric properties of AUQEI are adequate for use among children with CP. However, there are some challenges with the “autonomy” factor (high data loss rates and low success rates regarding the internal consistency of the items) and also with the reliability of the internal consistency of the factors that need to be taken into consideration.

The percentage of data loss was high for the “autonomy” factor. The items with the highest frequency of blank responses were those that asked what the child’s feelings were in the following: “when you get marks at school” (item 24; “autonomy” factor), “when you sleep away from home” (item 17; “autonomy” factor”), “when you play with your brother or sister” (item 3; “family” factor) and “when you have to stay in the hospital” (item 14). It is possible that most of these questions are not pertinent for children with an incapacitating neurological disease like CP because of the limitations imposed by the disease. None of the items remained unanswered by the healthy children in the present study, and this was also seen in the only study that calculated this rate among children with sickle-cell anemia [34].

The reliability was generally seen to be adequate. Only the factor “autonomy” had results that were lower than desired with regard to the internal consistency of the items. This analysis had only previously been performed in one study, on children with sickle-cell anemia, which found inadequate values for the “family” and “leisure” factors [34]. The variation in these results reinforces the need for verification of the psychometric properties for each group studied, which should be done before interpreting the results relating to QL[33],[35].

The reliability of the internal consistency was assessed as adequate in the general analysis of the instrument, and was similar to other studies[13], [14], [34],[36]. However, the reliability for each factor was insufficient. Although most authors have only used the total AUQEI score for interpreting the results[15], [14],[34],[37], some authors have taken factor scores into consideration in evaluating QL[38],[39]. For this to be done, Cronbach’s alpha coefficient has to be reapplied to each factor before interpreting the results.

The validity was shown to be generally adequate for the characteristics tested. Only the “functioning” factor had moderate correlations with the “family” and “autonomy” factors in the evaluation of the discriminating validity. Similar results have been found in other studies[14],[38], which may suggest that these factors are measuring similar constructs, or that one aspect of QL may be interfering mutually in one or more factors.

The construct validity was assessed as adequate, since AUQEI enables discrimination between children with CP and healthy children. Other studies using AUQEI have also found differences in the perception of QL when comparing healthy children with children presenting a chronic disease [34],[37], with the exception of one study on autistic children [39].

The convergent validity was shown to be adequate only for the AUQEI “functioning” factor as it had significant correlation with the CHQ-PF 50 physical summary. However, there were no correlations between the AUQEI and the CHAQ disability index or the CHQ-PF 50 psychosocial summary. We believe the results found had occurred, in part, due to limitation of the choice of instrument.

First of all, the CHQ-PF 50 and the CHAQ have been validated to be answered by parents and AUQEI is answered by the children. Some studies have shown that, in general, children and adolescents with CP tend to perceive a minor impact on their QL compared with the perception of their parents[40],[41], there is a low concordance between the perception of children with CP and that of their parents [41],[42]. The judgment of children in this age group and the manner in which they assign values may contain particularities, as there may be crucial differences between what isconsidered a quality “infant” life from the perspective of an adult and that of the child [43]. Although we know that, at the time the study was conducted, there were no instruments available to the Brazilian population to be answered by children.

The second point about the limitation of the construct validity is due to the nature of the instruments used. AUQEI is a generic QL instrument designated to evaluate what is more important in the point of view of the child, how satisfied he/she is about his/her wishes and hopes. On the other hand, the CHQ-PF 50 is a generic instrument of health-related quality of life (HRQOL) and the CHAQ is a specific instrument to assess HRQOL. HRQOL refers to the satisfaction and well-being of an individual with regard to their physical, psychological, social, economic and spiritual development relative to their state of health[44]. As only the AUQEI “functioning” factor presents a question related to health condition (“how do you feel when you go to a medical evaluation?”), it explains the correlation found with the physical summary of the CHQ-PF 50. But this question does not exactly correspond to the incapacity measured by the CHAQ instrument, so there was no correlation found with the CHAQ.

It was the first time that the convergent validity of AUQEI was tested and it can best be evaluated in future studies using instruments answered by children. Unfortunately, it will be difficult to find an instrument that is as conceptually appropriate to the infant universe as AUQEI.

The low frequencies of floor and ceiling effects suggests that this instrument is adequate for evaluating and discrimination between individuals with different clinical conditions, which in fact was seen in evaluating the construct validity.

The present study should be interpreted taking into consideration possible methodological limitations. The sample size was reduced because more than one third of the eligible patients presented cognitive deficits. However, the proportion of children with CP and intellectual impairment (intelligence quotient [IQ] <70) ranges from 40% to 67.6% in the literature [45].Our findings were near this range (38.9%). On the other hand, this exclusion enabled an important degree of methodological rigor, given that the application of the instrument has to be conditional on the child’s cognitive capacity to comprehend the questions.

According to the literature, the more severe motor impairments are associated with the largest intellectual dysfunctions [46]. The lowest levels of intelligence in children with CP are presented in individuals with higher deficits in gross motor function (GMFCS level-V) and spastic quadriplegia [45]. Thus, the instrument was not applied to and is not capable of evaluating patients with greater degrees of impairment. This is an unbridgeable limitation in QL studies on this population.

This study presents statistical power, since according to the calculation of sample size performed from the prevalence of CP (2.18/1000 live births[29], equal to 0.218%), a confidence level of 99% and a margin of error of 1.5%, the sample size is 45 individuals, which is the number of individuals who completed the questionnaire.

Another methodological aspect to be noted is the fact that the interviews of the children were conducted by a psychologist and we placed a lot of importance on ensuring that the children were comfortable in order to understand and answer the questionnaire. Unfortunately the time expended to complete the questionnaire was not measured.

New validation studies on AUQEI should be conducted, and factorial analysis should be added in order to identify possible problems, particularly with regard to the “autonomy” factor[13].

In conclusion, AUQEI was shown to be a reliable and valid instrument for evaluating children with CP when its total score was used. The reliability was insufficient to use the factors separately. Convergent validity should continue to be tested in future studies.

## References

[1] Davis E, Shelly A, Waters E, Davern M. (2010) Measuring the quality of life of children with cerebral palsy: comparing the conceptual differences and psychometric properties of three instruments. Dev Med Child Neurol 52(2): 174–80. 10.1111/j.1469-8749.2009.03382.x 19549193

[2] Carlon S, Shields N, Yong K, Gilmore R, Sakzewski L, et al (2010) Asystematic review of the psychometric properties of Quality of Life measures for school aged children with cerebral palsy. BMC Pediatrics 10: 81 Available: http://www.biomedcentral.com/1471-2431/10/81 10.1186/1471-2431-10-81 21059270 PMC2995480

[3] World Health Organization Quality of Life (WHOQOL) (1997) Measuring quality of life. Available: http://www.who.int/mental_health/media/68.pdf. Accessed 2014 March 15.

[4] Bax M, Goldstein M, Rosenbaum P, Leviton A, Paneth N, et al (2005) Proposed definition and classification of cerebral palsy, April 2005. Dev Med Child Neurol 47(8): 571–576. 16108461 10.1017/s001216220500112x

[5] Akodu AK, Oluwale OA, Adegoke ZO, Ahmed UA, Akinola TO (2012) Relationship between spasticity and health related quality of life in individuals with cerebral palsy. Nig Q J HospMed 22(2): 99–102. 23175906

[6] Morales NMO, Silva CH, Frontarolli AC, Araújo RR, Rangel VO, et al (2007) Psychometric properties of the initial Brazilian version of the CHQ-PF50 applied to the caregivers of children and adolescents with cerebral palsy. Qual Life Res 16(3): 437–444. 17334831 10.1007/s11136-006-9136-6

[7] Shikako-Thomas K, Dahan-Oliel N, Shevell M, Law M, Birnbaum R, et al (2012) Play and be happy? Leisure participation and quality of life in school-aged children with cerebral palsy. Int J Pediatr 10.1155/2012/387280 22919403 PMC3420102

[8] Dey M, Landolt MA, Mohler-Kuo M (2012) Health-related quality of life among children with mental disorders: a systematic review. Qual Life Res 21(10): 1797–814. 22298200 10.1007/s11136-012-0109-7

[9] Upton P, Lawford J, Eiser C (2008) Parent-child agreement across child health-related quality of life instruments: a review of the literature. Qual Life Res 17(6): 895–913. 10.1007/s11136-008-9350-5 18521721

[10] Varni JW, Burwinkle TM, Berrin SJ, Sherman SA, Artavia K, et al (2006) The PedsQL in pediatric cerebral palsy: reliability, validity, and sensitivity of the Generic Core Scales and Cerebral Palsy Module. Dev Med Child Neuro l48(6): 442–449. 16700934 10.1017/S001216220600096X

[11] Dickinson H, Parkinson K, McManus V, Arnaud C, Beckung E, et al (2006) Assessment of data quality in a multi-centre cross-sectional study of participation and quality of life of children with cerebral palsy. BMC Public Health 6: 273–285. 17087828 10.1186/1471-2458-6-273 PMC1636041

[12] Waters E, Davis E, Mackinnon A, Boyd R, Graham HK, et al (2007) Psychometric properties of the quality of life questionnaire for children with CP. Dev Med Child Neurol 49: 49–55. 17209977 10.1017/s0012162207000126.x

[13] Manificat S, Dazord A (1997) Évaluation de la qualité de vie de l’enfant: validation d’un questionnaire, premiers résultats. Neuropsychiatr Enfance Adolesc 45(3): 106–114.

[14] Assumpção FB Jr, Kuczynski E, Sprovieri MH, Elvira MG (2000) Escala de avaliação de qualidade de vida (AUQEI—AutoquestionnaireQualité de Vie Enfant Imagé): validade e confiabilidade de uma escala para qualidade de vida em crianças de 4 a 12 anos. Arq Neuropsiquiatr 58(1): 119–127. 10770876 10.1590/s0004-282x2000000100018

[15] Barbosa APM, Zampa TN, Iwabe C, Diz MAR (2012) Relação da Sobrecarga do Cuidador, Perfil Funcional e Qualidade de Vida em Crianças com Paralisia Cerebral. Rev Neurocienc 20(30): 367–371.

[16] Borges MBS, Werneck MJS, da Silva ML, Gandolfi L, Pratesi R (2011) Therapeutic effects of a horse riding Simulator in children with cerebral palsy. Arq Neuropsiquiatr 69(5): 799–804. 22042184 10.1590/s0004-282x2011000600014

[17] Hodgkinson I, D’Anjou MC, Dazord A, Berard C (2002) Qualité de vie d’une population de 54 enfants infirmes moteurs cérébraux marchants. Éstude transversale. Ann Readapt Med Phys 45(4): 154–158. 11960659 10.1016/s0168-6054(02)00195-2

[18] Wechsler D (2002) WISC III—Escala de inteligência Wechsler para crianças. 3.ed São Paulo: Casa do Psicólogo 10.1016/j.ophtha.2014.10.028

[19] Russell DJ, Rosenbaum PL, Cadman DT, Gowland C, Hardy S, et al (1989) The gross motor function measure: a means to evaluate the effects of physical therapy. Dev Med Child Neurol 31(3): 341–352. 2753238 10.1111/j.1469-8749.1989.tb04003.x

[20] Palisano R, Rosenbaum P, Walter S, Russell D, Wood E, et al (1997) Developmental and reliability of a system to classify gross motor function in children with cerebral palsy. Dev Med Child Neurol 39(4): 214–223. 9183258 10.1111/j.1469-8749.1997.tb07414.x

[21] Sankar C, Mundkur N. (2005) Cerebral Palsy–Definition, Classification, Etiology and Early Diagnosis. Indian JPediatr 72: 865–868. 16272660 10.1007/BF02731117

[22] Scholtes VAB, Becher JG, Beelen A, Lankhorst GJ. (2006) Clinical assessment of spasticity in children with cerebral palsy: a critical review of available instruments. Dev Med Child Neurol 48: 64–73. 16359597 10.1017/S0012162206000132

[23] Green LB, Hurvitz EA. (2007) Cerebral Palsy. Phys Med RehabilClin N Am 18: 859–882. 17967366 10.1016/j.pmr.2007.07.005

[24] Brasil: Ministério da Saúde. (2013) Diretrizes de Atenção à pessoa com Paralisia Cerebral. Brasília-DF. Available: http://bvsms.saude.gov.br/bvs/publicacoes/diretrizes_atencao_paralisia_cerebral.pdf. Accessed 2014 February 20.

[25] Landgraf JM, Maunsell E, Speechley KN, Bullinger M, Campbell S, et al (1998) Canadian–French, German and UK versions of the Child Health Questionnaire: Methodology and preliminary item scaling results. Qual Life Res 7(5): 433–445. 9691723 10.1023/a:1008810004694

[26] Morales NMO, Silva CHM, Frontarolli AC, Araújo RRH, Rangel VO, et al (2007) Psychometric properties of the initial Brazilian version of the CHQ-PF50 applied to the caregivers of children and adolescents with cerebral palsy. Qual Life Res 16(3): 437–444. 17334831 10.1007/s11136-006-9136-6

[27] Machado CSM, Ruperto N, Silva CHM, Ferriani VP, Roscoe I, et al (2001) The Brazilian version of the childhood health assessment questionnaire (CHAQ) and the child health questionnaire (CHQ). ClinExpRheumatol 19(Suppl 23): S25–S29. 11510326

[28] Morales NMO, Funayama CA, Rangel VO, Frontarolli AC, Araújo RR, et al (2008) Psychometric properties of the child health assessment questionnaire (CHAQ) applied to children and adolescents with cerebral palsy. Health Qual Life Outcomes 6: 109 10.1186/1477-7525-6-109 19055820 PMC2631579

[29] Himmelmann K, Uvebrant P. (2014) The panorama of cerebral palsy in Sweden. XI. Changing patterns in the birth-year period 2003–2006. Act Paediatr 103(6): 618–624. 10.1111/apa.12614 24575788

[30] Centro de Estudos, Pesquisas e Projetos Econômico-Sociais (CEPES). (2003) Uberlândia—Painel de informações municipais: painel comemorativo de 40 anos do Instituto de Economia-UFU e 25 anos do CEPES. Available: http://www.portal.ie.ufu.br/cepes/tabelas/Painel/Painel2003.pdf Accessed 2014 Sep 10.

[31] Cochran WG. (1997) Sampling techniques. 3. ed New York: John Wiley & Sons, 428p.

[32] McHorney CA, Ware JE Jr, Lu JF, Sherbourne CD (1994) The MOS 36-item short-form health survey (SF-36): III. Test of data quality, scaling assumptions, and reliability across diverse patient groups. Med Care, 32(1): 40–66. 8277801 10.1097/00005650-199401000-00004

[33] Health outcomes methodology symposium (2000) Glossary. Med Care 38(9)(Suppl 2): 7–13. 10982107 10.1097/00005650-200009002-00031

[34] Ferreira PRA, Pinto RMC, Morales NMO, Silva CHM (2008) Propriedades psicométricas do AutoquestionnaireQualité De Vie Enfant Imagé (AUQEI) aplicado em crianças com doença falciforme. Hor Ci 2(1): 47–69.

[35] McCarthy ML, Silberstein CE, Atkins EA, Harryman SE, Sponseller PD, et al (2002) Comparing reliability and validity of pediatric instruments for measuring health and well-being of children with spastic cerebral palsy. Dev Med Child Neurol 44(7): 468–476. 12162384 10.1017/s0012162201002377

[36] Manificat S, Dazord A, Cochat P, Nicilas J (1997) Évaluation de la qualité de vie en pédiatrie: comment recueillir le point de vue de l’enfant. Arch Pediatr 4(12): 1238–1246. 9538430 10.1016/s0929-693x(97)82616-4

[37] Kuczynski E, Silva CA, Cristófani LM, Kiss MH, Odone Filho V, et al (2003) Quality of life evaluation in children and adolescents with chronic and/or incapacitating diseases: a Brazilian study. AnPediatr (Barc) 58(6): 550–555. 12781110 10.1016/s1695-4033(03)78120-x

[38] Barreire SG, Olcinei AO, Kazama W, Kimura M, Santos VLCG (2003) Qualidade de vida de crianças ostomizadas na ótica das crianças e das mães. J Pediatr (Rio J) 79(1): 55–62. 12973510

[39] Elias AV, Assumpção FB Jr. (2006) Qualidade de vida e autismo. Arq Neuropsiquiatr 64(2a): 295–299. 16791372 10.1590/s0004-282x2006000200022

[40] White-Koning M, Arnaud C, Dickinson HO, Thyen U, Beckung E, et al (2007) Determinants of child-parent agreement in quality-of-life: a European study of children with cerebral palsy. Pediatrics 120(4): e804–814. 17908738 10.1542/peds.2006-3272

[41] Gates P, Otsuka N, Sanders J, McGee-Brown J. (2010) Functioning and health-related quality of life of adolescents with cerebral palsy: self versus parent perspectives. Dev Med Child Neurol 52(9): 843–849. 10.1111/j.1469-8749.2010.03666.x 20477836

[42] Varni JW, Burwinkle TM, Sherman SA, Hanna K, Berrin SJ, et al (2005) Health-related quality of life of children and adolescents with cerebral palsy: hearing the voice of the children. Dev Med Child Neurol 47(9): 592–597. 16138665

[43] Eiser C. (1997) Children’s quality of life measures. Arch of DisChild 77(4): 350–354. 9389244 10.1136/adc.77.4.350 PMC1717360

[44] Guyatt GH, Naylor CD, Juniper E, Heyland DK, Jaeschke R, et al (1997) Users’ guides to the medical literature. XII. How to use articles about health-related quality of life: evidence-based medicine working group. JAMA 277(15): 1232–1237. 9103349 10.1001/jama.277.15.1232

[45] Dalvand H, Dehghan L, Hadian MR, Feizy A, Hosseini SA. (2012) Relationship Between Gross Motor and Intellectual Function in Children With Cerebral Palsy: A Cross-Sectional Study. Arch Phys Med Rehabil 93: 480–484. 10.1016/j.apmr.2011.10.019 22265344

[46] Vilibor RHH, Vaz RH. (2010) Correlação entre a função motora e cognitiva de pacientes com Paralisia Cerebral. Rev Neurocienc 18(3): 380–385.

